# Management of a High-Risk Surgery with Emicizumab and Factor VIII in a Child with a Severe Hemophilia A and Inhibitor

**DOI:** 10.1055/s-0041-1728667

**Published:** 2021-05-12

**Authors:** Charles R. Lefèvre, Anaïs Jaffré, Adeline Pontis, Fabienne Nedelec-Gac, Pierre Guéret, Isabelle Gouin-Thibault, Bernard Fraisse, Sophie Bayart, Benoit Guillet

**Affiliations:** 1 Laboratoire d'Hémostase Bioclinique, CHU de Rennes, Bretagne, France; 2 Centre Régional de Traitement de l'Hémophilie, CHU de Rennes, Bretagne, France; 3 Univ Rennes, CHU Rennes, Inserm, EHESP, Irset (Institut de recherche en santé, environnement et travail) - UMR_S 1085, Rennes, France; 4 Service de Réanimation Pédiatrique, CHU de Rennes, Bretagne, France; 5 Service de Chirurgie Pédiatrique, CHU de Rennes, Bretagne, France

The recent development of a humanized, bi-specific, and monoclonal antibody mimicking the function of activated factor VIII was a revolution in the management of patients suffering from severe hemophilia A with inhibitors. 1 The phase III randomized studies have shown a more efficient prophylaxis of this subcutaneous administered drug in these patients compared with recombinant FVIIa (rFVIIa) and activated prothrombin complex concentrates (aPCC). 2 3 Nonetheless, there are “real life” matters that need to be explored in this new era of managing hemophilia patients, such as surgery management under emicizumab, especially in children. 4 5 6 Here, we report the first case, to our knowledge, of major orthopedic surgery managed with factor VIII infusions in a child with inhibitor receiving emicizumab.

**Fig. 1:**
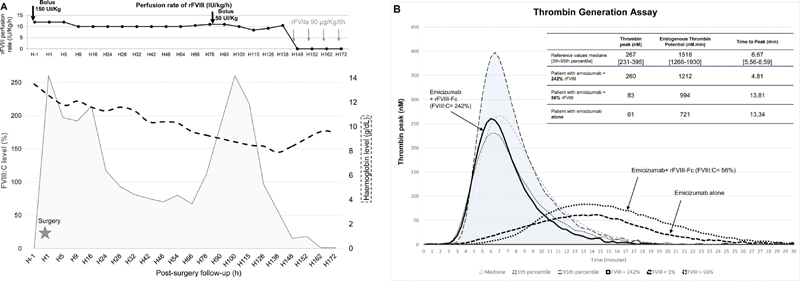
( **A** ) Evolution of FVIII:C and haemoglobin levels. ( **B** ) Evolution of thrombin generation assays. Reference values were established in 32 healthy patients.

Our patient is a 9-year-old boy with severe hemophilia A. He presented an inhibitor after the 10th exposed day with a factor VIII (FVIII) concentrate (historic peak: 412 BU/mL). Thereafter, he presented an important intracranial hemorrhage (subdural and intraparenchymal hematomas). The first treatment with rFVIIa (Novoseven, 90 µg/kg/2 h) and tranexamic acid was inefficient. It was therefore switched with aPCC (FEIBA 80 IU/kg/8 h) that was rapidly effective. He also presented some knees and left hip hemarthrosis responsible for arthropathies. Several attempts of immune tolerance induction through Port-a-Cath failed whatever the FVIII concentrate used, recombinant FVIII or plasma-derived FVIII. Because of an insufficient efficacy of rFVIIa for totally stopping some bleeding events, a prophylaxis with aPCC was performed with daily infusion of 80 IU/kg. Given the cumbersome nature of this treatment, emicizumab (Hemlibra) was introduced instead of aPCC in December 2018. Emicizumab was started with 3 mg/kg/week for 4 weeks and then adapted to 1.5 mg/kg/week. The boy had no more bleeding including clinically evident hemarthrosis. However, the left hip arthropathy worsened with aggravation of limping and pain leading to the use of a wheelchair. Hip X-ray showed an osteonecrosis of the left femoral head requiring a femoral varization osteotomy that was planned in June 2020. Because the inhibitor titer was low at 2 BU/mL, rFVIII-Fc (Elocta) was administered to normalize the coagulation during the surgery. A rFVIII-Fc bolus with 6,000 IU (150 IU/kg) was initially administered, followed by a continuous infusion 12 IU/kg/h ( Fig. 1A ). The emicizumab treatment was continued (1.5 mg/kg/week).

This complex surgery performed on rFVIII-Fc required a close monitoring with frequent FVIII:C measures to adjust rFVIII-Fc administrations. All FVIII:C levels were measured with a chromogenic method (STA-R Max3 analyzer ; TriniCHROM FVIII:C reagent – Stago) by using bovine coagulation factors X and IXa that do not interfere with emicizumab. 7 8 9 In same plasma samples, thrombin generation assays (TGA) were performed by using low concentration of tissue factor (1 pM) as suggested for the TGA in hemophilia A patients. 10 TGA were performed before the surgery without rFVIII-Fc and throughout rFVIII-Fc infusions ( Fig. 1B ).

**Fig. 2:**
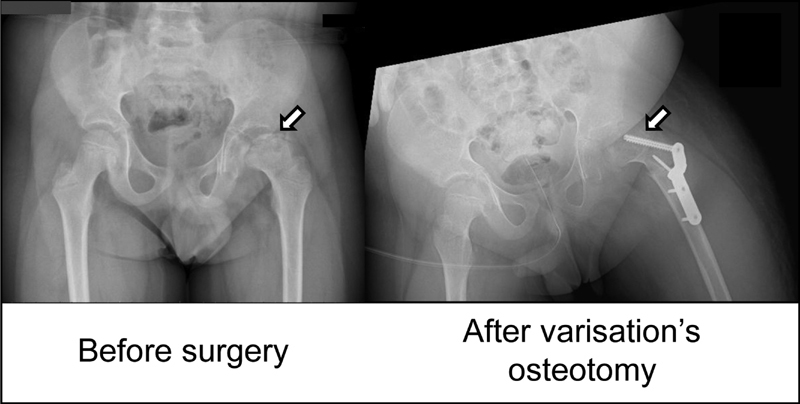
The arrow on the left shows the severe arthropathy of the left hip with a coxa plana and an alteration of cartilageneous and osseous structures. The arrow on the right shows the osteosynthesis material inserted after the varisation osteotomy of the femoral neck.

The FVIII:C level measured 30 minutes after bolus was high at 242% and maintained above 200% during the first 24 hours, following postsurgery without change of the rFVIII-Fc infusion rate ( Fig. 1A ). The orthopedic surgery included a proximal femoral derotation osteotomy and then fixed by osteosynthesis material ( Fig. 2 ). No bleeding (or other complication) occurred during and after surgery with low per-operatory blood loss (200 mL). His immediate postsurgery hemoglobin level was 11.8 g/dL. After the first postsurgery day, the FVIII:C levels progressively decreased until 100% at H28. At the 78th hour, a 50 IU/kg rFVIII-Fc bolus was performed for drains ablation. After a new peak, the FVIII:C level further decreased despite maintaining the continuous infusion at the dose of 10 IU/kg/h because of the inhibitor resurgence at H162 with a titer of 1.8 BU/mL which rose to 33 BU/mL at H200. TGA's parameters evolved within normal ranges during first postsurgical 24 hours and then progressively decreased together with FVIII:C levels ( Fig. 1B ). The 6th postsurgery day, rFVIII-Fc was replaced with rFVIIa boluses at 90 µg/kg that were stopped after 1 day. During his stay, the patient did not bleed and therefore did not require any red blood cell concentrate transfusion; the lowest hemoglobin he presented was 7.9 g/dL and was only corrected with a single iron sucrose injection (Venofer). Furthermore, no markers of coagulation activation were detected during rFVIII-Fc and rFVIIa treatments. Always receiving a prophylaxis with emicizumab alone, the patient was able to walk within 2 months after surgery and presented no bleeding.

To date, most of the surgery reports performed with emicizumab in cases with inhibitor, concerned adults. rFVIIa was most used to prevent bleeding during and after surgical procedures. In pediatrics, only minor procedures were described. 4 5 6 11 12 13 Our case is so, to our knowledge, the first description of a major orthopaedic surgery managed with FVIII concentrates in a child with severe hemophilia A with inhibitor while receiving a prophylaxis with emicizumab. The choice we made of using rFVIII-Fc for bleeding prevention during and after the surgery was foremost driven by the presence of a low titer inhibitor. Furthermore, we prohibited aPCC because of its thrombotic risk in association with emicizumab. 14 Finally, rFVIIa was ruled out because it was often only partially effective for this patient. We show here that, as for some adults reported to date, major surgeries can be safely managed with FVIII concentrates in children with severe hemophilia A with inhibitor while receiving emicizumab. This dual treatment could also be applicable without an inhibitor at the doses usually administered. 15 In our patient, the FVIII:C chromogenic assay appeared to be a reliable tool for peri-surgical monitoring in children despite concomitant treatment with emicizumab as it was proposed. 8 9 TGA with low TF concentration performed in parallel to FVIII:C measures could be helpful. 9 However, our results did not show a perfect correlation between TGA's parameters and normalized FVIII levels. The TGA with these conditions is so not enough reliable for the monitoring of emicizumab treatments and need further adjustments. Finally, it was rapidly observed that maintaining the prophylaxis with emicizumab reduces the duration of post-surgical treatment in major orthopaedic surgeries. 13 Indeed, for our patient, as already reported for adults receiving emicizumab, FVIII or rFVIIa injections were only necessary until the 7th day postsurgery. Thereafter, prevention with emicizumab alone was sufficient to protect against late bleeding during rehabilitation.
